# Rethinking imaging in axial spondyloarthritis: toward diagnostic maturity

**DOI:** 10.1016/j.ero.2026.100182

**Published:** 2026-05-13

**Authors:** Manouk de Hooge, Gaëlle Varkas, Anna Moltó, Sofia Ramiro

**Affiliations:** 1 Department of Rheumatology, Ghent University Hospital, Ghent, Belgium; 2 Department of Rheumatology, Leiden University Medical Center, Leiden, The Netherlands; 3 Center for Inflammation Research, Molecular Immunology and Inflammation Unit, VIB- Ghent University, Zwijnaarde, Belgium; 4 Paris Cité University, UFR Medicine, Paris, France; 5 Department of Rheumatology, Bichat Hospital, APHP.Nord, Paris, France; 6 Université Paris Cité and Université Sorbonne Paris Nord, Inserm, INRAE, Centre for Research in Epidemiology and Statistics (CRESS), Paris, France; 7 Zuyderland Medical Center, Heerlen, The Netherlands

## Abstract

Imaging, particularly magnetic resonance imaging (MRI) scans of the sacroiliac joints, has become central in the evaluation of axial spondyloarthritis (axSpA), partly due to the absence of reliable biomarkers and variable human leukocyte antigen B27 (HLA-B27) prevalence. However, its increasing use as a standalone diagnostic tool represents a conceptual shift that risks undermining clinical reasoning. This viewpoint critically examined the specificity and limitations of imaging findings and highlighted that lesions such as bone marrow oedema and structural changes may also occur in non-SpA populations, including healthy individuals and those with mechanical back pain. Misinterpretation, rather than intrinsic imaging limitations, is a major driver of diagnostic error.

In this viewpoint it is argued that imaging should serve a supportive and prognostic role, integrated within a broader clinical framework that includes patient history, clinical features, and laboratory data. Overreliance on imaging contributes to diagnostic inflation, particularly in non-adiographic and atypical populations, with implications for patient care and healthcare systems. Emerging tools such as artificial intelligence may amplify these challenges if not carefully validated.

Recentring clinical reasoning, promoting multidisciplinary collaboration, and ensuring imaging is requested and interpreted within an appropriate clinical context are essential steps forward. Ultimately, imaging should inform but not replace clinical judgement in the diagnosis of axSpA.

## INTRODUCTION

Axial spondyloarthritis (axSpA) is a chronic inflammatory disease characterised by inflammation of the sacroiliac joint (SIJ) and spine. Patients typically present with chronic back or buttock pain, often accompanied by prolonged morning stiffness. Chronic back pain is one of the most common complaints in primary and first-line care. In early axSpA, extra-musculoskeletal manifestations that would support the diagnosis are often absent, making early clinical recognition challenging.

Moreover, only about one-third of patients with axSpA show an elevated level of C-reactive protein (CRP), which has contributed to an increasing reliance on imaging in the diagnostic process. In the absence of reliable biomarkers and given the variable prevalence of human leukocyte antigen B 27 (HLA-B27) across populations, imaging—a magnetic resonance imaging (MRI) scan in particular—is often treated as a standalone criterion. It is important to note that most diagnostic decisions rely on an SIJ MRI scan, although a spine MRI scan alone rarely suffices for diagnosis. Nevertheless, this shift has fundamentally altered the diagnostic landscape: imaging is no longer used primarily to support a clinical suspicion but is increasingly treated as the sole tool for establishing or ruling out an axSpA diagnosis.

This approach is misguided, as imaging findings alone cannot make a diagnosis. It is rather a supportive tool that must always be interpreted in the context of the clinical picture. Reliance on imaging alone for diagnostic decision-making may increase the risk of error and undermine clinical reasoning, with potential adverse effects on patient care.

## SPECIFICITY AND LIMITATIONS OF IMAGING FINDINGS IN axSpA

Inflammatory lesions on MRI scan, primarily bone marrow oedema (BME), capsulitis, and enthesitis, are typically associated with axSpA. However, much of the apparent non-specificity of BME arises from misinterpretation, inappropriate use, or lack of clinical context, rather than limitations of the MRI scan itself. Studies have demonstrated BME presence in several non-SpA populations, including postpartum women [[1], [2], [3], [4]], athletes [1,5,6], and most importantly for differential diagnosis, patients with mechanical chronic back pain [1,[7], [8], [9]]. Moreover, MRI scan lesions in both the SIJ and spine have been reported in healthy asymptomatic individuals, with frequency increasing with age [7,10,11].

Structural lesions such as ankylosis/new bone formation, erosions, fat lesions, and sclerosis—hallmarks of long-standing axSpA—are likewise not immune to misinterpretation when considered outside their clinical context and may also be identified in non-SpA contexts [10,12]. To complicate interpretation further, emerging data highlight sex-specific patterns in anatomical variants and in the spatial distribution of lesions [13].

Historically, before the conceptualisation of nonradiographic (nr-) axSpA, radiographic (r-) axSpA was considered a predominantly male disease. This led to a research base dominated by male cohorts, with findings subsequently extrapolated to the broader axSpA spectrum. As a result, the female axSpA phenotype, including the pelvic anatomy and imaging features in both health and disease, has been largely overlooked [14]. Longitudinal work in postpartum women has demonstrated that both nonspecific BME and structural MRI scan abnormalities do not seem to evolve substantially over 5 years. Therefore, these lesions seem to follow a distinct trajectory compared with axSpA, adding a time dimension to imaging interpretation and reinforcing the need for cautious, contextualised interpretation [15].

## THE PROPER ROLE OF IMAGING

Undeniably, imaging holds considerable value in diagnosis and disease monitoring and should be integrated into clinical decision-making alongside clinical assessment. An MRI scan allows sensitive visualisation of inflammatory lesions, providing clinicians with information on both the presence and extent of inflammation, including depth and localisation. It remains indispensable for assessing baseline structural damage and monitoring structural progression. Additionally, the evolution of lesions over time can assist in refining diagnostic probability. Its proper role, however, is prognostic and supportive, not determinative, and final diagnostic decisions remain the responsibility of the rheumatologist, who must integrate imaging findings with clinical features and other data.

For example, an MRI scan lesion scoring system, such as the Spondyloarthritis Research Consortium Canada (SPARCC) scoring, is useful in research settings to measure and quantify response to treatment. For example, rigorously applying the Assessment of SpondyloArthritis International Society (ASAS) definition of a positive MRI scan may misclassify individuals with mechanical stress-related lesions as having axSpA. Such illustrative cases underscore that numerical thresholds alone should not replace diagnosis.

These challenges underscore the importance of using imaging judiciously, with interpretation guided by experienced radiologists or readers whose expertise is crucial in distinguishing true disease from incidental findings, especially as novel technologies constantly reshape the field. The adoption of artificial intelligence (AI) is rapidly expanding, with growing applications in rheumatology, including automated lesion assessment in imaging. These emerging tools promise major advances in lesion detection, characterisation, and differentiation. However, if applied without caution, they risk opening Pandora’s box as current AI models are trained on human interpretation, which is, by default, imperfect. This reinforces the need for careful use of AI in both clinical and research contexts and for the development of future models with iterative cycles of training, validation, and refinement to ensure robustness and accurate performance.

## IMAGING IS NOT A CLINICAL DIAGNOSIS

Imaging abnormalities alone cannot be equated with a diagnosis of axSpA. Findings visible on an MRI scan, radiography, or computed tomography (CT) scan may or may not represent pathology, and they may or may not be clinically relevant. Labelling such changes as ‘diagnostic’ is misleading. In the absence of a clinical suspicion of axSpA, imaging alone cannot determine the diagnosis of axSpA [16]. The diagnostic process in axSpA is, and must remain, rooted in clinical reasoning and evaluation.

The diagnosis of axSpA arises not from a single investigation but from the overall gestalt of the disease. This gestalt is anchored in the clinical picture: a young adult patient with chronic back pain, often with features of inflammatory back pain; the presence of HLA-B27; elevated inflammatory markers such as CRP; and possible imaging findings that support the diagnosis. Additional SpA features or family history may further strengthen clinical suspicion, but it is this combination of parameters—rather than any single one in isolation—that characterises a patient with axSpA. Since the introduction of the ASAS classification criteria, there has been a growing, but incorrect, tendency to apply them as if they were diagnostic criteria. This checkbox-style approach has led to clinicians ticking off features instead of embedding them in clinical reasoning. Consequently, imaging tends to dominate the diagnostic landscape due to the increasing trust in medical technology, overshadowing traditional clinical assessment and undermining diagnostic accuracy. Moreover, an indecisive or completely normal MRI scan does not exclude axSpA in patients with a convincing clinical phenotype, including inflammatory back pain, and should not in itself preclude diagnosis.

### The problem of imaging-driven overdiagnosis

The reported prevalence of axSpA has risen markedly in recent years [17]. Although disease awareness and early recognition partly explain these findings, much of the increase reflects imaging-driven inflation, especially in nonradiographic, female, HLA-B27-negative patients [18]. The broader use of MRI scans has created diagnostic noise: incidental lesions, ambiguous findings, and overinterpretation of physiological changes. The result is diagnostic pollution: a surge of supposed cases that do not reflect true disease. Not only does this result in exposure to inadequate, potentially harmful treatment, but it also burdens healthcare systems and distorts epidemiologic data. Most of all, it lengthens the patient journey to the correct diagnosis. Although improved recognition has undoubtedly helped identify underdiagnosed patients, the balance remains delicate, and the equilibrium may now be shifting unfavourably. Without pretest and posttest reasoning, imaging creates confusion rather than clarity.

### Path forward: refinement, not rejection

There is a call for refinement, where clinical reasoning is recentred. Imaging should be requested only within an appropriate clinical context, and if available before evaluation, it should be interpreted only after a thorough clinical assessment. A comprehensive differential diagnostic framework must be maintained, considering all contextual factors, such as mechanical strain, degenerative disease, infection, and (multi)parity.

Further, multidisciplinary collaboration between radiologists and rheumatologists is essential, with radiologists’ expertise central for accurate imaging finding descriptions aiding interpretation, ensuring the contextualisation of findings. The combined experience of both specialists is critical for accurate diagnosis and informed decision-making and minimising overinterpretation of incidental findings [19]. Finally, as imaging remains important and our understanding of its role in the field continues to evolve, criteria involving imaging should be both updated and respected. Classification systems must maintain a careful balance across multiple domains, including clinical, laboratory, and imaging findings.

This principle is reflected in recent developments. The updated ASAS-SPARTAN classification criteria for axSpA, presented at the American College of Rheumatology Convergence 2025, broaden the definition of positive imaging to include both active inflammatory (BME) and structural lesions of the SIJ. Although structural lesions are rare in early disease and isolated BME is not highly specific, their combined assessment could improve diagnostic specificity when interpreted alongside compatible clinical features [20].

The revision of the classification criteria was based on data from the Classification of Axial SpondyloarthritiS Inception Cohort (CLASSIC) study, which validated a weighted set of imaging and clinical variables to reach prespecified sensitivity and specificity goals, with final results exceeding the thresholds (sensitivity 79.5% and specificity 90.4%), emphasising the role of imaging within a holistic clinical framework [21].

Although this viewpoint is focused on axSpA, the above principles apply across medicine. There is a cultural drift towards overreliance on advanced diagnostic techniques, such as an image-first diagnosis. Technical investigations, such as imaging, are often considered more objective or reliable than the clinical encounter and are sought prematurely as shortcuts to perceived diagnostic certainty. As we have shown, this approach can be problematic, as images are a means to an end, not the indisputable truth. If clinical reasoning is removed from the equation, the practice of medicine itself is diminished.

## CONCLUSION

In axSpA, imaging is playing an indispensable role in diagnosis and disease assessment, but its findings must be interpreted within a broader clinical framework. Imaging abnormalities, even when highly suggestive, are most meaningful when contextualised by clinical reasoning rather than used in isolation. Moving forward, we must pair clinical vigilance with imaging literacy. Hence, the maturity of the use of imaging in the field will be measured not by the number of scans performed, but by the ability to integrate them into a nuanced clinical framework. The future of axSpA diagnosis rests on context, restraint, and careful integration. This means that imaging should support the clinical picture and inform, but never override, the rheumatologist’s clinical judgement. Recognising this distinction is essential for maintaining diagnostic accuracy.

## Editor disclosure

The peer review process did not involve Editorial Board Member Sofia Ramiro, and the editorial decision-making was led by editors who were not involved in the creation of this manuscript.

## Funding

This research did not receive any specific grant from funding agencies in the public, commercial, or not-for-profit sectors.

## CRediT authorship contribution statement

**Manouk de Hooge:** Writing – review & editing, Writing – original draft, Investigation, Data curation, Conceptualization. **Gaëlle Varkas:** Writing – review & editing. **Anna Moltó:** Writing – review & editing. **Sofia Ramiro:** Writing – review & editing.

## Competing interests

MDH is a speaker or has received consultancy fees from UCB Pharma; MDH is also a Director of MDH Research. GV is a speaker or has received consulting fees from AbbVie, Amgen, Alfasigma, UCB, Pfizer, Eli Lilly, Novartis, Johnson & Johnson, Galapagos, and Celltrion. Research support: Takeda, Celltrion, Galapagos, AbbVie, MSD, and EG. AM has received consultancy and speaker fees from AbbVie, Alfasigma, Eli Lilly, Johnson & Johnson, MSD, Novartis, Pfizer, and UCB, and research grants from AbbVie and UCB. SR has received consultancy fees from AbbVie, Alfasigma, Eli Lilly, Johnson & Johnson, MSD, Novartis, Pfizer, and UCB; speaker fees from Eli Lilly, Novartis, and UCB; and research grants from AbbVie, Alfasigma, Eli Lilly, MSD, Novartis, Pfizer, and UCB.
